# A formal model of interpersonal inference

**DOI:** 10.3389/fnhum.2014.00160

**Published:** 2014-03-25

**Authors:** Michael Moutoussis, Nelson J. Trujillo-Barreto, Wael El-Deredy, Raymond J. Dolan, Karl J. Friston

**Affiliations:** ^1^Wellcome Trust Centre for Neuroimaging, University College LondonLondon, UK; ^2^Brain Dynamics Department, Cuban Neuroscience CentreHavana, Cuba; ^3^School of Psychological Sciences, University of ManchesterManchester, UK

**Keywords:** free energy, active inference, value, evidence, surprise, self-organization, interpersonal, Bayesian

## Abstract

**Introduction:** We propose that active Bayesian inference—a general framework for decision-making—can equally be applied to interpersonal exchanges. Social cognition, however, entails special challenges. We address these challenges through a novel formulation of a formal model and demonstrate its psychological significance.

**Method:** We review relevant literature, especially with regards to interpersonal representations, formulate a mathematical model and present a simulation study. The model accommodates normative models from utility theory and places them within the broader setting of Bayesian inference. Crucially, we endow people's prior beliefs, into which utilities are absorbed, with preferences of self and others. The simulation illustrates the model's dynamics and furnishes elementary predictions of the theory.

**Results:** (1) Because beliefs about self and others inform both the desirability and plausibility of outcomes, in this framework interpersonal representations become beliefs that have to be actively inferred. This inference, akin to “mentalizing” in the psychological literature, is based upon the outcomes of interpersonal exchanges. (2) We show how some well-known social-psychological phenomena (e.g., self-serving biases) can be explained in terms of active interpersonal inference. (3) Mentalizing naturally entails Bayesian updating of how people value social outcomes. Crucially this includes inference about one's own qualities and preferences.

**Conclusion:** We inaugurate a Bayes optimal framework for modeling intersubject variability in mentalizing during interpersonal exchanges. Here, interpersonal representations are endowed with explicit functional and affective properties. We suggest the active inference framework lends itself to the study of psychiatric conditions where mentalizing is distorted.

## Introduction

There is growing interest in modeling behavioral and physiological responses with biologically grounded normative models, particularly in emerging disciplines such as neuroeconomics and computational psychiatry. The motivation for these developments rests upon characterizing behavioral phenotypes in terms of underlying variables that have a principled functional and—in some instances—neurobiological interpretation. Recently, optimal decision making has been formulated as a pure inference problem to provide a relatively simple (active inference) framework for modeling choice behavior and inference about hidden states of the world generating outcomes (Friston et al., 2013). This is a potentially important development because it provides a coherent and parsimonious (Bayes) optimal model of behavior. This normative model is consistent with classical treatments, such as expected utility theory and softmax response rules, without calling on *ad hoc* parameters like inverse temperature or temporal discounting. This means that, in principle, one can characterize people's behavior in terms of prior beliefs about the world (as well as the confidence or precision of those beliefs).

In this paper, we demonstrate that this approach can also be applied fruitfully when choices—and the underlying preferences—are based upon interpersonal beliefs about oneself and other people. Social cognition merits special analysis as it presents substantial challenges. An active inference framework can usefully address some of these, but not without new theoretical considerations. In what follows, we describe the sorts of beliefs that may underlie interpersonal exchange and use simulations of active inference to demonstrate the behaviors that ensue. In subsequent work, we hope to use these simulated choices to explain observed behavior so as to characterize subjects in terms of model parameters that encode interpersonal beliefs. The routines used for the simulations of this paper are available as part of the academic SPM freeware and can be adapted to a variety of games.

### Theories of affectively charged beliefs about self and others

*Self*- and other- representations often are heavily affect-laden and a vast literature is devoted to them. We cannot do justice to this entire field here and instead focus on four groups of theories about interpersonal representations. Firstly, “homeostatic” theories hold that an adequately positive self-representation is so important in itself that healthy humans will even sacrifice accurate explanations of social and psychological events to maintain positive self-representations. Classic psychological-defense theories (Ogden, 1983; Rycroft, 1995) and attribution theories (Bentall, 2003) fall into this group. These theories easily explain the biases that healthy people and psychiatric patients exhibit in seeing the self in rosy colors (e.g., grandiosity) or others in negative colors (e.g., racism) as self-representation-boosting manoeuvres. Hence, these theories also explain how the motives for one's behavior can be ulterior to the motives that the agent believes they are acting under. However, experimental support for these theories is incomplete (Moutoussis et al., 2013). Secondly, economic theories usually consider one's true preferences as known to the agent; while at the same time their behavior may be directed at instrumentally managing their reputation vis-a-vis others, including deceiving them (Camerer, 2003). Some social-psychological theories combine these utilitarian perspectives into one construct, social desirability, said to have both self-deceit and image-management components (Crowne and Marlowe, 1960). Thirdly, there are a group of theories that see many adult beliefs about the self and others as products of learnt information-processing, relatively divorced from current interests. Examples are the rigid “core beliefs” that people often hold about themselves according to some cognitive-behavioral theories (Waller et al., 2001) or the inaccurate beliefs formed when strong affects are said to overwhelm peoples' ability to think about their own mind and that of others (Allen et al., 2008). Finally, there are theories that take into account both the fluidity and uncertainty of person-representations (like many clinical and psychological theories) and an *explicit, current* functional role for them (like the neuroeconomic tradition). This is a smaller tradition, exemplified by the “sociometer theory of self-esteem” (Leary et al., 1995). Here a particular aspect of self-representation—self-esteem—predicts whether other people are likely to include or exclude one from social interactions. As access to human (e.g., friends, partners), material (e.g., work opportunities), safety and other resources can be dramatically reduced by social exclusion, self-esteem helps predict the success of social interactions. When it comes to other-representation, the “sinister attribution error” theory of apparently unwarranted suspiciousness (Kramer, 1994) formalizes a somewhat similar logic: that taking others to be less well-meaning than they are serves to minimize false-negative errors in the detection of social difficulties. However, the theories of Leary et al. and of Kramer are qualitative, insufficiently general, and have not been applied to interactive exchanges.

We seek to generalize the “sociometer theory” to encompass *all* self- and other- representations that can be reasonably inferred within interpersonal exchanges. In this paper, we provide a specific computational example of this. Psychologically it is easy to appreciate how making inferences about others helps to make predictions: For example, “a fair person will not exploit me.” Similarly about the self, “honest people like me are trusted.” However, interpersonal representations may come to serve as preferred outcomes themselves; for example, “I'd prefer to be a fair person and to deal with fair people.” They may summarize (and even hide) social, cultural and ultimately evolutionary goals that are not otherwise explicitly represented.

### Computational challenges of decision-making in social exchanges

One might expect people to maximize the overt benefits that they extract from social interactions, such as food or mates, by logically thinking through different policies and choosing the best. However, such a project faces serious challenges, of which we consider three. These motivate using interpersonal representations to make predictions about exchanges and active inference to infer both representations and policies.

The first challenge concerns the potentially *explosive complexity* of social cognition. As a key example, interpersonal cognition is recursive. In order to achieve maximum material benefit I need to predict how another person will react. To do this I need to imagine what they will decide. However, they should do the same—estimate what I intend to do. Therefore I have to estimate what they think that I intend to do. But they will do the same and so on, without a well-defined end. In contrast, real people in real situations only perform a very limited number of such recursive steps. We argue that using interpersonal beliefs can increase the effective depth of (otherwise costly) cognition.

The second concerns the *arbitrariness of the parameterization* of many decision-making schemes. As a central example, just one parameter is often used to describe the precision (inverse noisiness) of choices given the values attached to these choices. This precision parameter is then fitted to observed choice behavior in an agnostic manner. The parameter in question has been interpreted in a number of ways that are almost impossible to distinguish: sometimes it is cast as intrinsic noise or error rate, implying that agents are incapable of more precise or deterministic choices. Sometimes, it is used to motivate a form of exploration, implying that there is something unknown about the situation and it is best not to put all one's eggs in one basket. At other times, it is seen as a sensitivity that reflects the change in behavior for a change in returns. This last interpretation is closely related to choice matching, whereby the preferred frequency of different outcomes is an increasing function of their utility and not a winner-takes-all preference. In learning paradigms it is also difficult to separate estimates of precision from the learning rate (Daw, 2011). Parameterization of an agents' choices in terms of a single noisiness parameter thus conflates error, exploration, choice matching and, in practice, learning rates.

The active inference framework addresses this problem first by taking account of the fact that there is always uncertainty about outcomes. In a probabilistic sense, optimal outcomes are better quantified in terms of probability distributions, as opposed to scalar reward or utility functions. We can then separate the optimal precision over action choice (en route to the outcome), which describes *how to best get to the desired distribution* over outcomes, from the *preferred outcome distribution itself*. The former precision can itself be optimized given beliefs about hidden states of the world and controlled transitions among them—through formulating choice behavior in terms of beliefs over policies. The precision in question is the precision of (or confidence in) beliefs about alternative policies. It still weighs the choice between different policies, but it is no longer a free parameter! In contrast the precision over outcome preferences is a *reward sensitivity*, in principle *testable independently of the task* at hand. In the active inference framework there is no need for a learning rate parameter as such—the optimal change of beliefs is inferred at each step.

The third computational challenge rests on *the difficult calculations entailed in using a model of the world* to draw inferences. Social inferences, for example, present a difficult inverse problem when disambiguating the meaning of a particular social datum: for example, “my partner gave me nothing” may be important both for self-representation (“maybe because I am worthless”) and for other-representation (“maybe because she is horrible”). The framework that we describe is well suited to deal with such ambiguities. Their resolution rests upon prior beliefs about social outcomes that can be updated on the basis of experience in a Bayes optimal fashion. This, like all statistical inversion of probabilistic models, is computationally challenging; the active inference framework suggests a practical solution based on so-called Variational Bayes (a ubiquitous instance of approximate Bayesian inference that finesses computational complexity). In this paper, we will use approximate Bayesian inference to show how interpersonal representations are accommodated in terms of prior beliefs; thereby providing a normative framework within which to parameterize different people and their interpersonal beliefs.

This paper comprises three sections. The first provides a brief introduction to active inference, with a special emphasis on how preferences and goals can be cast in terms of prior beliefs about eventual outcomes. This enables goal-directed behavior to be described purely in terms of inference about states of the world and subsequent behavior. The second section introduces a Trust game to illustrate the formal aspects of modeling interpersonal exchanges within this framework. The third (Results) section uses simulations of this game under active inference to highlight how interpersonal beliefs produce characteristic choice behaviors. We conclude with a discussion of putative applications of this approach to normative behavioral modeling.

## Methods

This section summarizes the building blocks of Active inference, which include the following: Adaptive agents are held to (i) set themselves desirable goals that they consider likely to achieve (ii) choose policies that maximize the likelihood of achieving these goals (iii) form beliefs about the world consistent both with their sensory observations and their goals. In this section, we also briefly describe a practical way of solving this inference problem, i.e., (iv) using an inference process that involves the passing of simple messages between cognitive modules. This Variational Bayes (VB) message passing or updating is a simpler and more biologically plausible method for performing approximate Bayesian inference than the schemes usually considered. We then formulate a model of a simple interpersonal exchange and describe its implementation so that others researchers can use it. The definition and meaning of the mathematical symbols we use is summarized in Table 1.

**Table 1:** **Additional definitions and significance of symbols that appear in equations**.

**Symbol**	**Definition and significance**	**Formula where symbol first appears**
*P*	Probability mass of a discrete random variable, or probability density of a continuous random variable	*P*(*s_T_*|*m*) = σ(*r*(*s_T_*), β)
*s_T_*	Outcome state—a state that the agent may arrive at time *T*, the end of the exchange	
*m*	Model of the world according to the agent. It includes all the rules of how the dynamics of the world evolve, as well as the parameters of the world that don't change as the world evolves	
β	Inverse temperature over outcomes. It signifies how strongly prior (utilitarian) beliefs change as a function of the outcome measure in question (e.g., money) at the point of indifference.	
σ ($\vec{x}$, β)	The Gibbs softmax function. It ascribes to each component of *x*_*i*_ ∈ $\vec{x}$ a probability proportional to exp(β*x_*i*_*)	
*r*(*x*)	Return associated with state *x*.	
*u_t_*, *ũ*	*u_t_* is a control state—that is, a state that the agent believes s/he will deploy at time t. In general this does not necessarily determine what action will be realized at time *t*—the agent may not have full control over this. However in our agents do have such control, so *u_t_* equates with the decision about the action to take. *ũ* is the sequence of control states believed to be taken from now to the outcome (e.g.,: “I will type in all the letters of my password”).	*ũ* = {*u*_*t*_ … *u*_*T*_}
γ	Precision of belief about control sequences. It signifies the confidence that the goal will be attained, if the best attainable combinations of control states are employed.	$\begin{array}{l} P(\tilde{u}|s_{t},\;\gamma ,\;m)=\\ =\frac{1}{Z}\text{exp}(-\gamma D_{KL}[P(s_{T}|s_{t},\;\tilde{u})||P(s_{T}|m)]) \end{array}$
*Z*	Normalizing constant. In many cases we consider how strong beliefs are relative to each other; Dividing each by their sum *Z* ensures they add up to one, as probabilities should.	
*D*_*KL*_[*P*_0_(*x*)||*P*_1_(*x*)]	Kullback-Leibler divergence between a distribution *P*_0_(*x*) and another distribution *P*_1_(*x*). It is the expectation *with respect to P*_0_(*x*) of the difference in surprise inherent in encountering each possible value of *x* according to the two distributions.	
Pr	Probability value; Pr(*x* = *a*) is the probability that *x* takes the value *a*.	P(õ, $\tilde{s}$, *ũ*, γ|*m*) = Pr({*o*_0_, …, *o*_*t*_} = õ, {*s*_0_, …, *s_t_*} = $\tilde{s}$, {*u_t_*, …, *u_T_*} = *ũ*, γ)
*P*(õ, $\tilde{s}$, *ũ*, γ|*m*)	Probability density according to the generative model *m*; i.e., the world including the agent	*P*(õ, $\tilde{s}$, *ũ*, γ|*m*) =
		= *P*(õ|$\tilde{s}$, *m*)*P*(*ũ*|*s*_*t*_, γ, *m*)*P*($\tilde{s}$|*m*)*P*(γ|*m*)
*Q*($\tilde{s}$, *ũ*, γ|μ)	*Q* is the belief that agent infers using the approximate inference scheme. Rather than being expressed in terms of probability distributions, it is expressed in terms of their “sufficient statistics” *μ*, such as its expectation. Not to be confused with ***Q***, the matrix representation of policy values.	*Q*($\tilde{s}$, *ũ*, γ|μ) ≈ Pr({*s*_0_, …, *s*_*t*_} = $\tilde{s}$, {*u*_*t*_, …, *u_T_*} = *ũ*, γ)
$\mu =({\overset{⌢}{s}}_{0},\;...\;,\;{\overset{⌢}{s}}_{t},\;\overset{⌢}{u},\;\overset{⌢}{\gamma })$	The specific instantiation of the sufficient statistics in our example.	$\mu =({\overset{⌢}{s}}_{0},\;...\;,\;{\overset{⌢}{s}}_{t},\;\overset{⌢}{u},\;\overset{⌢}{\gamma })$
*H*[*P*(*x*)]	*H* is the Entropy of the distribution *P*(*x*). It is a measure of the average surprise of this distribution	−*D*_*KL*_[*P*(*s*_*T*_|*s*_*t*_, *ũ*)||*P*(*s*_*T*_|*m*)] =
		= *H*[*P*(*s*_*T*_|*s*_*t*_, *ũ*)] + *E*_*P*(*s*_*T*_|*s*_*t*_, *ũ*)_[ln *P*(*s*_*T*_|*m*)]
*E*_*P*(*x*)_[ln *P*(*x*)]	*E*_*P*(*x*)_[*f*(*x*)] signifies the Expectation of *f*(*x*) under the probability distribution *P*(*x*). In active inference ln *P*(*x*) is a measure of utility.	

### Summary of active inference

#### Setting plausible goals

In active inference, action elicits outcomes that are the most plausible under beliefs about how they are caused. This approach contrasts with normative formulations in optimal decision theory, where actions are chosen to maximize the value of outcomes rather than plausibility. However, beliefs about outcomes are not motivationally neutral—an agent believes that her actions will lead to *good* outcomes. Therefore, if the prior beliefs about outcomes—the agent's goals or hopes—reflect the utility of those outcomes, then active inference can implement optimal policies, effectively seeking out the outcomes with the greatest utility.

In general, agents may have subtle reasons to distribute their prior beliefs over particular outcomes. They may, for example, use a matching law such as Herrnstein or softmax mapping to preserve ecological resources or to distribute goods among conspecifics. We model an agent's preference with a softmax function σ(*r*(*s*_*T*_), β) of objective returns *r* at the outcome time *T*, so that prior (utilitarian) beliefs for any agent or model *m*, are written as follows:

(1) $$P(s_{T}|m)=\sigma (r(s_{T}),\;\beta )$$

This describes a probability distribution over states *s*_*T*_ at time *T*. Probability depends upon the return associated with each state. This classical utility function is expressed as a map from objective ultimate outcomes to prior beliefs, with the relative utility of different outcomes depending upon a sensitivity parameter *β*.

#### Choosing policies to achieve the plausible goals

Suppose that an agent believes that at time *t* they occupy a state *s_t_*. They then need to choose a policy comprising a sequence of control states *ũ* = {*u*_*t*_ … *u*_*T*_} that leads to the desired outcome distribution *P*(*s*_*T*_|*m*). If *ũ* leads to a distribution over final or outcome states *P*(*s*_*T*_|*s*_*t*_, *ũ*), then success can be measured by the Kullback-Leibler divergence between the anticipated and desired distribution. The agent can then choose policies according to this measure of their likely success. Following Friston et al. (2013), we can express this formally as follows:

(2) $$P(\tilde{u}|s_{t},\;\gamma ,\;m)=\frac{1}{Z}\text{exp}(-\gamma D_{KL}[P(s_{T}|s_{t},\;\tilde{u})||P(s_{T}|m)])$$

Here, we have introduced a normalizing constant *Z* and a confidence or precision parameter *γ*. While the softmax parameter β in Equation 1. calibrates the relative utility of different outcomes, the precision parameter *γ* encodes the confidence that desired goals can be reached, based on current beliefs about the world and the policies available. Unless otherwise stated we will use the unqualified term “precision” for *γ*. Crucially, precision has to be inferred so that the confidence is optimal, in relation to the current state (context) and beliefs about the current state and future states.

#### Forming beliefs consistent with observations and goals

In our model, agents need to perform inference about certain quantities. An agent's knowledge of how they interact with the world can be expressed as a joint distribution over these requisite quantities:

(3) $$P(\tilde{o},\;\tilde{s},\;\tilde{u},\;\gamma |m)=\mathrm{Pr}(\{o_{0},\;\ldots ,\;o_{t}\}=\tilde{o},\;\{s_{0},\;\ldots ,\;s_{t}\}=\tilde{s},\;\{u_{t},\;\ldots ,\;u_{T}\}=\tilde{u},\;\gamma )$$

This probabilistic knowledge constitutes a generative model over observations, states, control and precision. This model is constituted by prior beliefs about policies *P*(*ũ*|*s*_*t*_, γ, *m*)—as specified by Equation 2—state transitions, the likelihood of a sequence of observations stemming from those states and prior beliefs about precision:

(4) $$P(\tilde{o},\;\tilde{s},\;\tilde{u},\;\gamma |m)=P(\tilde{o}|\tilde{s},\;m)P(\tilde{s}|\tilde{u},\;m)P(\tilde{u}|s_{t},\;\gamma ,\;m)P(\gamma |m)$$

Agents can use this model to infer the hidden states of the world $\tilde{s}$ = {*s*_0_ · · · *s*_*t*_}; to determine where each policy, or sequence of choices, *ũ* = {*u*_*t*_ · · · *u*_*T*_}, is likely to lead; and to select the precision *γ* that encodes the confidence in policy selection. Agents can infer hidden states, their policy and precision from observed outcomes by inverting the model above. To do this they have two assets at their disposal: their observations *õ* = {*o*_1_ · · · *o*_*t*_} and their model *m* of choice-dependent probabilistic state transitions.

To keep things simple, we assume a one-to-one mapping between observations and states of the world. This is encoded by an identity matrix **A** with columns corresponding to states, rows corresponding to observations and elements encoding the likelihood of observations—*P*(õ|$\tilde{s}$, *m*), under their model.

#### State transitions in an interpersonal world

We can describe the possible states of the world as a cross product between a subspace which is hidden and one which can be observed. An example of the former is “my partner is cooperative” whereas an example from the latter is “they will give me nothing.” We model transitions between hidden states as constrained by the meaning of these subspaces. The part of the world-state that describes my partner's traits cannot change (otherwise they would not be traits). The part which describes their actions will be a probabilistic function of what I will do. As an example, the action “they will give me nothing” is probable if I follow a policy of giving them nothing myself.

Agents therefore describe changes in the world contingent upon what they do in terms of a 3-D transition matrix. This matrix ***B***(*u_t_*) has one “page” for each control state *u_t_* that the agent can employ. Each page has columns of possible states at time *t*; and rows of the possible states at time *t+1*. The entries of ***B*** are the probabilities *P*(*s*_*t*+1_|*s*_*t*_, *ũ*). As the reader may have noticed, the policy-dependent probabilities in Equation 2 can be derived by the repeated application of ***B***.

#### A practical method for performing inference

If agents have at their disposal a function *F* that approximates how *in*consistent their beliefs and observations were, they can minimize *F* to maximize the chance of achieving their goals. A suitable function *F* is the free energy of observations and beliefs under a model of the world. The reader is referred to Friston et al. (2013) for a full explication of free energy in active inference. For our purposes, we just need to know that *F* provides a measure of the probability of the observations under the model *F* ≈ −ln *P*(õ|*m*). This means that minimizing free energy renders observations the least surprising, under my model: “Given that I am likely to be at work in an hour (belief under model of the world) it is not surprising that I'm in a train station (observation); it *would be surprising* if I headed for the cinema (belief about behavior).” The free energy defined by a generative model is thus an objective function with respect to optimal behavior—where optimality is defined by the agent's beliefs.

Posterior beliefs correspond to an *approximate posterior probability* over states, policies and precision. These beliefs are parameterized by sufficient statisticsμ ∈ ℝ^*d*^ such that *Q*($\tilde{s}$, *ũ*, γ|μ) ≈ Pr({*s*_0_, …, *s*_*t*_} = $\tilde{s}$, {*u*_*t*_, …, *u*_*T*_} = *ũ*, γ). The free energy then becomes a function of the sufficient statistics of the approximate posterior distribution. This allows us to express approximate Bayesian inference in terms of free energy minimization:

(5) $$\mu_{t}=\arg{\text{min}}_{\mu }F(\tilde{o},\;\mu )$$

where actions or choices are sampled from Pr (*a_t_* = *u_t_*) = *Q*(*u_t_*|μ_*t*_). This means policies are selected that lead to the least surprising actions and outcomes. In summary, the optimization of sufficient statistics (usually expectations) rests upon a generative model and therefore depends on prior beliefs. It is these beliefs that specify what is surprising and consequently optimal behavior in both a Bayesian and utilitarian (optimal decision theory) sense.

**Figure 1:**
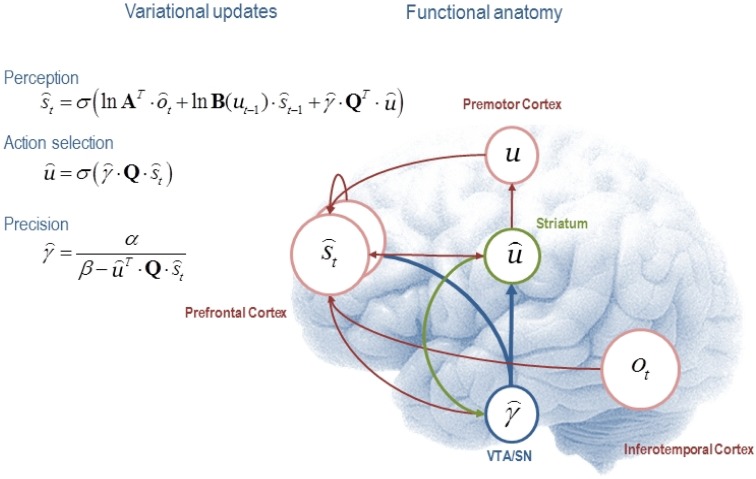
**This figure illustrates the cognitive and functional anatomy implied by the mean field assumption used in Variational Bayes**. Here, we have associated the variational updates of expected states with perception, of future control states (policies) within action selection and, finally, expected precision with evaluation. The updates suggest the sufficient statistics from each subset are passed among each other until convergence to an internally consistent (Bayes optimal) solution. In terms of neuronal implementation, this might be likened to the exchange of neuronal signals via extrinsic connections among functionally specialized brain systems. In this (purely iconic) schematic, we have associated perception (inference about the current state of the world) with the prefrontal cortex, while assigning action selection to the basal ganglia. Crucially, precision has been associated with dopaminergic projections from ventral tegmental area and substantia nigra. See Friston et al. (2013), whence this figure has been adapted, for a full description of the equations.

A common scheme used to perform free-energy minimization is VB. Many statistical procedures used in everyday data analysis can be derived as special cases of VB. We will not go into technical details and interested readers can find a treatment of VB relevant to the present discussion in Friston et al. (2013). Here, we note that VB allows us to partition the sufficient statistics into three common-sense subsets: statistics describing beliefs about states of the world causing observations; statistics describing beliefs about the (future) policy *ũ* = {*u*_*t*_ … *u*_*T*_} to be selected; and statistics describing beliefs about precision γ: $\mu =({\overset{⌢}{s}}_{0},\;...\;,\;{\overset{⌢}{s}}_{t},\;\overset{⌢}{u},\;\overset{⌢}{\gamma })$. These statistics are updated with each new observation, using variational message passing (VMP). Each belief (about precision, about the state of the world etc.) is a probability distribution held in a “node” of a network of such beliefs, as in Figure 1. Each belief not only has a most-likely-value but also an uncertainty, and possibly other features, that describe the exact shape of the distribution. In our case, these features are encoded by the statistics above. In VMP, the belief distributions and their associated parameters (sufficient statistics) are chosen from amongst a rich and flexible—but not unlimited—vocabulary, the so-called conjugate-exponential belief networks. When one of the beliefs—say, the sensory state—is updated via an observation, it is no longer consistent with the others: the free energy increases. The “node” of the network representing this belief then sends information about its new content (e.g., the expectation or mean of the distribution) to all the other belief “nodes” with which it is connected. It also sends information about the beliefs on which it depends to nodes sending messages, which mandates a reciprocal or recurrent message passing. The recipient “nodes” then adjust their parameters, and thus change the beliefs they encode, so as to increase consistency with the source of the message. Of course, this may put them a little out of line with yet other beliefs. Hence messages propagate back and forth via all the connections in the network, changing the statistical parameters that the nodes hold, until free energy cannot be reduced any further and consistency is once again optimized (Winn and Bishop, 2005).

The simplicity and generality of this VMP scheme speaks to the biological plausibility of its neuronal implementation (Friston et al., 2013). A common objection to Bayesian schemes is that it is implausible that the brain performs long algebraic derivations, or alternatively high-dimensional numerical integration, every time a new task was at hand. However, evolution may have converged on the simplicity and efficiency of VMP—or at least something like it.

Figure 1 shows the architecture of variational updates for any generative model of choice outcomes and hidden states that can be formulated as a Markov decision process. The functional anatomy implied by the update equations is shown (schematically) on the right. Here the distributions over observations given hidden states are categorical and parameterized by the matrix **A** as above. Similarly, the transition matrices ***B*(***ut***)** encode transition probabilities from one state to the next, under the current control state of a policy (*ũ* = {*u*_*t*_ · · · *u*_*T*_}).

In the simulations that follow we used a prior over precision that has a gamma distribution with shape and scale parameters *α* = 8 and *θ* = 1. The matrix ***Q*** contains the values of the i-th policy from the j-th current state and corresponds to the divergence term in Equation 2. We see that expectations about hidden states of the world are updated on the basis of sensory evidence, beliefs about state transitions and value expected under allowable policies. Conversely, policies are selected on the basis of the expected value over hidden states, while precision is monotonically related to value expected over hidden states and policies. See Friston et al. (2013) for details.

### Inferences about people in a model task

#### The simplified trust game

To illustrate the basic features of this formulation we construct a model^1^ of a simplified Trust Task based on the multi-round Investor-Trustee game (King-Casas et al., 2008). I (*self*) am to play consecutive rounds with the Trustee (*other*). At each round *t* I earn a wage *w*^*self*^, usually set at 20 units of play money. I can then invest one of a discrete set of fractions *f*^*self*, *low*^, …,*f*^*self*, *high*^ of my wage in a joint venture with the *other*. The investment is multiplied by a gain *g*, representing the surplus value created by the joint venture (usually *g* = 3). The *other* then returns a fraction of the *invested* amount. The round ends with the following returns:

(6) $$\begin{array}{l} \;r_{t}^{self}=w^{\;self}-w^{\;self}f^{self}+w^{\;self}f^{self}f^{other}\\ r_{t}^{other}=w^{\;self}f^{self}g-w^{\;self}f^{self}f^{other} \end{array}$$

Our Trust-Task is simpler than the standard Investor-Trustee game, with respect to the levels of investment and repayment available to the players. We make available only two levels, thus rendering a matrix representation of the exchange more straightforward and allowing experimenters to enforce (psychologically) interesting choices. The available response fractions *f* correspond only to *Cooperation* (action 1) or *Defection* (action 2). A matrix of monetary returns for *self* and *other* that can be used for this simplified task is shown in Table 2.

**Table 2:** **Trust Task monetary returns matrix with only two choices for each partner**.

	**Other**	
*self*	*u*^*o*^ = 1	*u*^*o*^ = 2
	(Cooperate: *f*^other, high^)	(Defect: *f*^*other, low*^)
*u*^*s*^ = 1	*r*^*s*^_11_ (e.g., = 26)	*r*^*s*^_21_ (e.g., = 10)
(Cooperate: *f*^*self*, *high*^)	*r*^*o*^_11_ (e.g., = 26)	*r*^*o*^_21_ (e.g., = 42)
*u*^*s*^ = 2	*r*^*s*^_12_ (e.g., = 21)	*r*^*s*^_22_ (e.g., = 18)
(Defect: *f*^*self*, *low*^)	*r*^*o*^_12_ (e.g., = 7)	*r*^*o*^_22_ (e.g., = 10)

*These returns are defined by payoffs r^s^_11_ > r^s^_12_ ≥ r^s^_22_ > r^s^_21_ for the self (in the Investor role) and r^o^_21_ > r^o^_11_ ≥ r^o^_22_ > r^o^_12_ for the other (in the Trustee role). The task is constructed as a sequential game, with my self playing first, and is typically asymmetric. In the example in brackets I have a “wage” of 20 units. I can choose to invest f^self, low^ = 20% or f^self, high^ = 80%; the other can choose to return 40 or 140% of my investment. All amounts have been rounded*.

The task is a multi-round game—partners have to make decisions, taking into account long-term consequences of their choices. This is a difficult problem—and we will see that appropriate use of interpersonal representations may be used as a shortcut.

#### Interpersonal representations and prosocial utilities

We now consider the issue of how preferences are constituted in the generative model. To construct our minimal model, we assume the following:

1. *self* and *other* are each represented by a single scalar quantity—“how good one is.” We will call this “esteem” so that *e*^*s*^ is how good the *self* is, while the esteem of the *other* is *e*^*o*^.
2. A “good” person, with positive esteem, is more likely to cooperate with an average person, all other things being equal.
3. An average person is more likely to cooperate with a “good” person, other things being equal.

The observable component of world states is disclosed by action (*u^o^*, *u^s^*) and the hidden component (*e^s^*, *e^o^*) concerns the traits to be inferred. The fact that a “good” person is more likely to cooperate—and to *attract cooperation*—highlights the fact that esteem can augment the utility of cooperation. An analogous reasoning applies to defection.

Preferential biases induced by esteem can be specified in terms of an augmented return that includes the payoff and esteem. Following the format of Table 2 we write:

(7) $$\begin{array}{l} r^{s}(u^{o}=1,\;u^{s}=1,\;e^{s},\;e^{o})=\beta_{r}^{s}r_{22}^{s}+e^{s}+e^{o}\\ r^{o}(u^{o}=1,\;u^{s}=1,\;e^{s},\;e^{o})=\beta_{r}^{o}r_{22}^{s}+e^{s}+e^{o} \end{array}$$

Table 3 gives the augmented returns for each combination of outcomes.

**Table 3:** **Utility matrix for the simplified Trust task**.

	**Other**	
*self*	*u*^*o*^ = 1 (Cooperate)	*u*^*o*^ = 2 (Defect)
*u^s^* = 1	β_*r*_*r*^*s*^_11_ + *e^s^* + *e^o^*	β_*r*_*r*^*s*^_21_ + *e^s^* + *e^o^*
(Cooperate)	β_*r*_*r*^*o*^_11_ + *e^s^* + *e^o^*	β_*r*_*r*^*o*^_21_ − *e^s^* − *e^o^*
*u*^*s*^ = 2	β_*r*_*r*^*s*^_12_ − *e^s^* − *e^o^*	β_*r*_*r*^*s*^_22_ − *e^s^* − *e^o^*
(Defect)	β_*r*_*r*^*o*^_12_ + *e^s^* + *e^o^*	β_*r*_*r*^*o*^_22_ − *e^s^* − *e^o^*

*The entries of Table 1 are weighted by a sensitivity parameter and then augmented by an interpersonal component to form socialized returns. The interpersonal component consists of the esteem for each partner plus the esteem for the other partner (weighted equally in this example). Positive esteems enhance cooperative utility whereas negative esteem increases the utility of defecting. We have assumed here that β^s^_r_ = β^o^_r_ = β_r_*.

With this setup observable outcomes can take just 5 values: A “starting state” and four outcomes: *o*_2_ = {*u*^*s*^ = 1, *u*^*o*^ = 1}, *o*_3_ = {*u*^*s*^ = 2, *u*^*o*^ = 1} and so on, for all combinations of cooperation and defection. For each round, each player has to model the transition probabilities *P*(*s*_*T*_|*s*_*t*_, *ũ*). If *r*^*o*^(*u*^*o*^_*t*_, *u*^*s*^_*t*_, *e*^*s*^, *e*^*o*^) denotes the augmented return for the *other*, *self* can use a softmax function to calculate the probabilities of actions taken by the *other* (following Equation 1):

(8) $$P(u_{t}^{o}|u_{t}^{s},\;e^{s},\;e^{o})=\frac{\text{exp}(r^{o})}{\sum_{c}\text{exp}(r^{o})}$$

However, this requires that *self* knows the beliefs of *other* about hidden esteems (*e^s^*, *e^o^*). We will assume that *self* uses beliefs about their esteem to model the beliefs of the *other*. We will see later that this is not an unreasonable assumption. Furthermore, we assumed that players can resolve just two levels of esteem *e*^*o*^ = *p* (for prosocial) or *e*^*o*^ = *n* (for non-social or antisocial). To further simplify things, we assume that the self esteem is neutral, *e*^*o*^ = 0. Prior beliefs about choices will then be influenced by “who I would like you to be” and “what I would like (us) to get.” These simplifications create a discrete hidden state space with 10 states. These correspond to the five observable states, for each of the two levels of the other's esteem *e*^*o*^ ∈ {*p*, *n*}. The action chosen by *self* were sampled from posterior beliefs over choices based on the prior beliefs over policies of Equation 2. These prior beliefs depended on the utilities in Table 3.

### Implementation of the trust game in iterated play

We implemented the multi-round version of the Trust game by using the posterior beliefs about the partner, at the end of each round, as the priors for the next round.

The software routines were written using the SPM academic freeware platform in matlab (MATLAB, 2012). The SPM platform, including the DEM toolbox used here, is available under GPL (GNU General Public License, version 3, 2007). It can be accessed via www.fil.ion.ucl.ac.uk/spm/software/spm12. Additional scripts are available from the corresponding author on demand, also under GPL.

## Results

**Figure 2:**
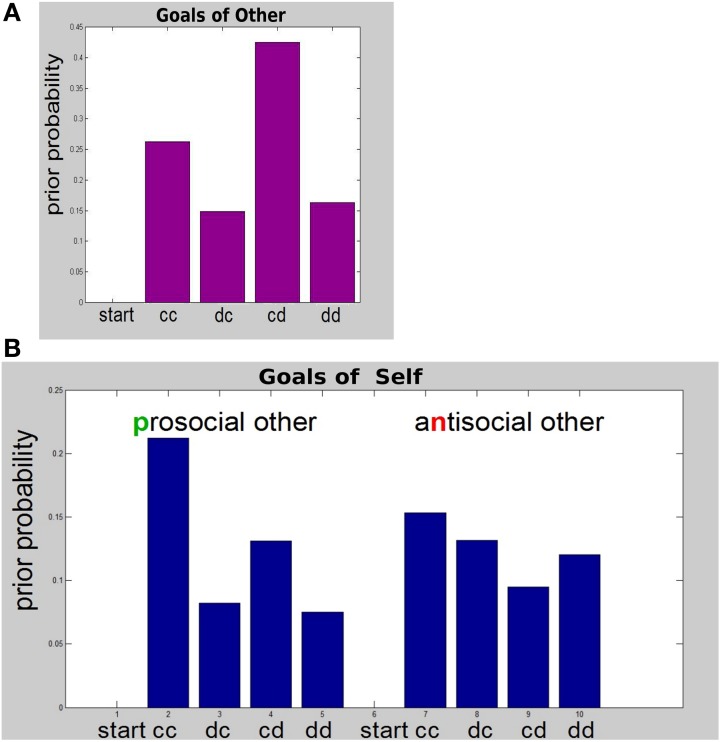
**Pattern of social utilities *P*(*s*_*T*|*m*_) = σ(*r*^*s*^ (*s*_*T*_), β)**. **(A)** Preferences of the *other*. This simple *other* only considers observable states of each round—the starting state (start), and each of the four *self*-action—*other*-action combinations shown in Table 3. The “start” state is only indicated for completeness: agents correctly never consider it as an outcome. **(B)** Preferences (goals) of the *self*. Preferences over all 10 hidden states are shown; See text for detailed description.

In order to perform simulations we used the monetary values in Table 1. To calculate the numerical values corresponding to Table 2, we chose a value for β_*r*_ such that the resulting probabilities according to Equation 8 would be very distributed. Furthermore, for the purposes of this demonstration, we chose the *other* to be antisocial; i.e., have a negative esteem, and naïve; i.e., only influenced by immediate outcomes (as per Equation 8). The preferences (priors) that these choices translate into for the *other* are shown in Figure 2A. The *other* would prefer the self to cooperate and the *other* herself to defect (cd in Figure 2A). Their second best preference would be mutual cooperation (cc), which still has a substantial monetary outcome. The *other* is indifferent about the remaining two options, in which the *self* defects (dc, dd). In Figure 2, we have included the starting state (start) as a potential outcome—as is required by the model specification in the code we used. We set the starting state probability to zero, as it never actually materializes as an outcome and agents do not need to consider a preference for it.

The situation is a little more complicated, and more interesting, with respect to the goals of the *self* that this scheme gives rise to. These are shown in Figure 2B. Whereas our antisocial, naïve *other* did not consider separate states for prosocial vs. antisocial self, we endowed the *self* with preferences depending on the type of the *other* and hence we consider the full 10-state outcome space for each round of the exchange.

Figure 2B shows that the preference of the *self* for mutual cooperation is more pronounced if the *other* is prosocial. As one might expect, given an antisocial *other* the second-best preference for *self* is for the *other* to cooperate while *self* defects. More interestingly, given a prosocial *other* the second-best preference for the *self* is to cooperate, while the prosocial *other* defects. Heuristically, *self* is forgiving toward prosocial but not antisocial *others*.

### A single-round

**Figure 3:**
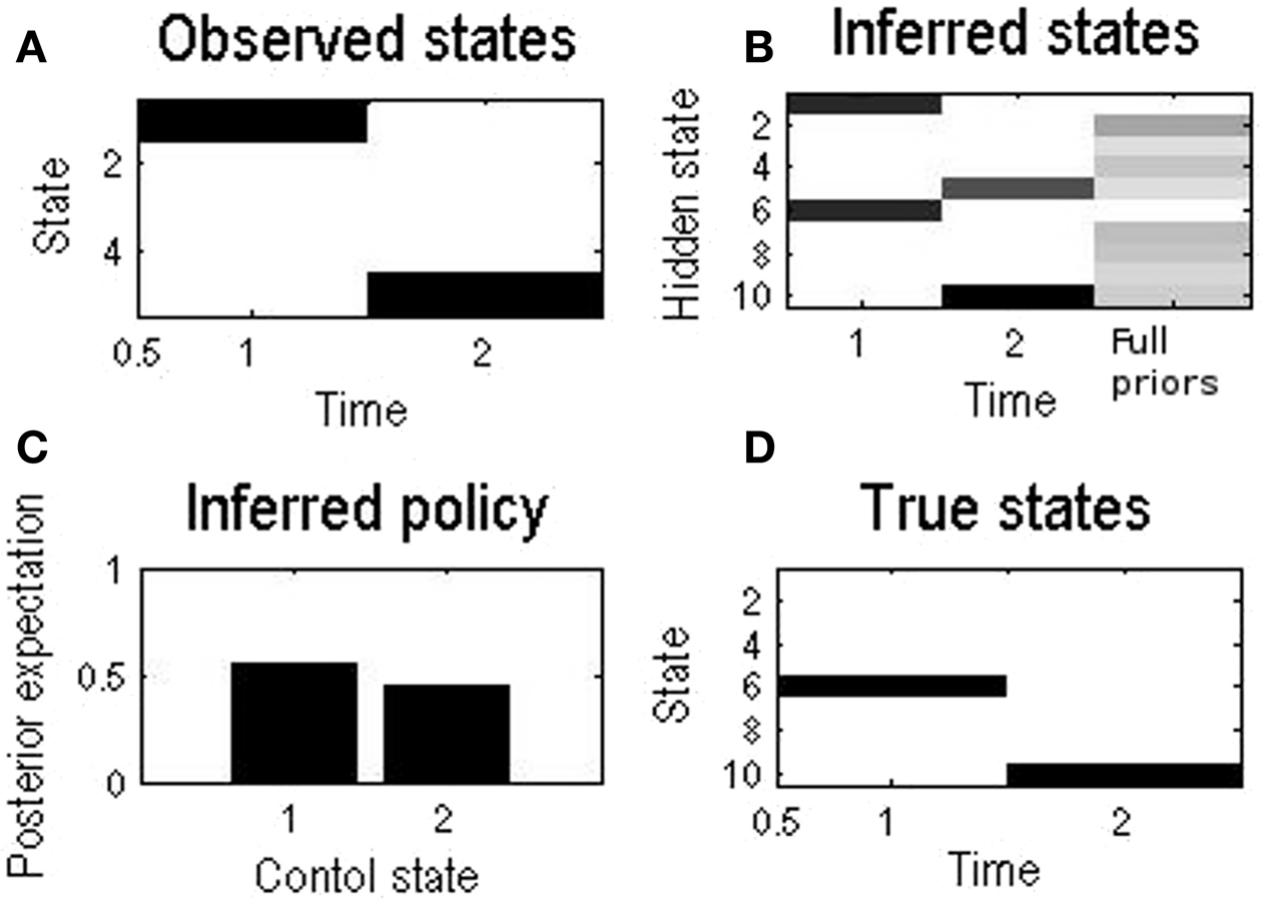
**Inferences made by *self* during a single round, where *self* initially believes that the *other* is just as likely to be prosocial as antisocial**. The numbering of states from 1 to 10 corresponds to the 10 states in Figure 2B. **(A)** This shows that the observable state changed from state 1, the starting state, to 5, corresponding to mutual defection during this example round. **(B)** Initially the belief of *self* was equally shared between playing a prosocial partner or an antisocial partner (state 1 or 6). At the end of the round, belief was shared between mutual defection with a prosocial (s5) or antisocial (s10) partner, but no longer equally so. Defection made the *self* infer that the *other* was more likely to be antisocial: *P*(s10) > *P*(s5). The column “Full priors” corresponds to Figure 2B. **(C)** Control state 1 (cooperation) is slightly favored despite agnosticism, at this stage, as to the type of the *other*. As it happened however the *self* still chose to defect, as choice is probabilistic **(D)**. The underlying true states: in this example the *other* is antisocial.

The basic behavior of *self* when choosing a policy through free energy minimization is shown in Figure 3. Initially, *self* believes that the *other* is equally likely to be *p* or *n*. In other words, at the beginning of a series of exchanges, we assume people are agnostic as to the character or esteem of their opponent. Notice that although there are 10 hidden states, there are only five observable states—because the esteem (of the other) is hidden and has to be inferred.

At the first time step *self* just observes the starting state and believes the *other* is equally likely to be *prosocial* or *antisocial*, corresponding to hidden states 1 or 6. Still, under the influence of their utilitarian priors *self* assigns a higher probability to the cooperative policy (control state 1). With the parameters used in this example, this is a modest preference: as it happens, the choice selected was to defect—to which the *other* responded by also defecting. *Self* therefore observes outcome state 5. Finally, on the basis of this outcome, *self* infers that they are more likely to be playing an *antisocial other*, which is the case. Clearly, in a single round, *self* cannot make use of this inference. However, if we now replace the prior beliefs about the *other* with the posterior beliefs and play a further round, we can emulate Bayesian updating of beliefs about the *other*. We now turn to the simulation of iterated play using this method of updating beliefs.

### Iterated play

**Figure 4:**
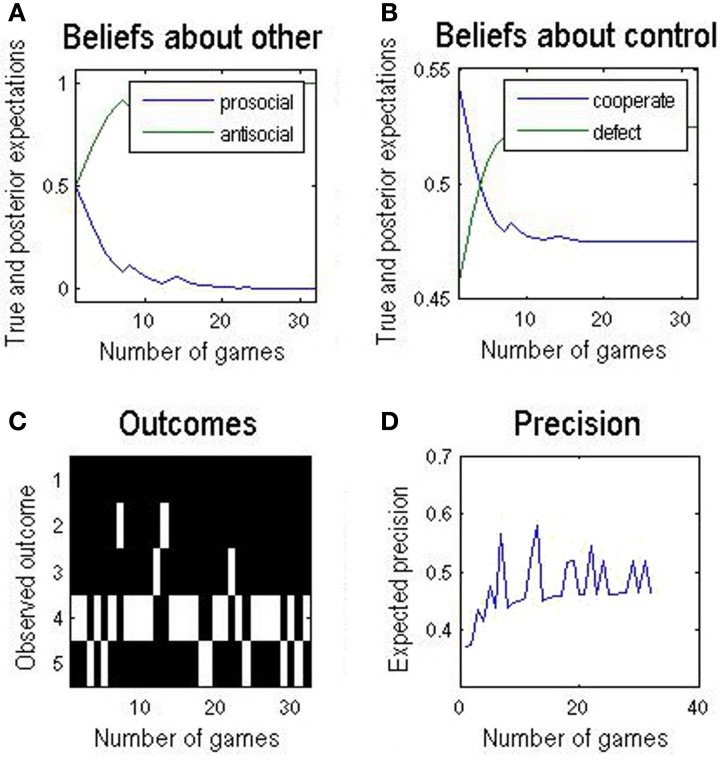
**(A)** A sequence of 32 rounds of the simplified Trust task. Over the course of approximately 10 rounds, *self* becomes confident that *other* is antisocial. **(B)** This increasing belief results in a declining belief in (preference for) cooperating. **(C)** In this example the actions chosen are quite variable and: **(D)** expected precision changes relatively slowly. The variability of responses is due to the relatively weak preferences over different outcomes used here; this is to illustrate how one quantity (e.g., expected precision) changes with respect to another (e.g., players' choices) over a single round or over a sequence of rounds.

During iterated play, beliefs about the *other* evolve. This has a knock-on effect on the goals or priors for each round—that produce a progressive change in preferred policies as one learns about the *other* and adjusts one's behavior accordingly. The result of a multi-round game is shown in Figure 4 and reveals several interesting features:

The agent infers fairly quickly that the *other* is antisocial and reduces cooperative play. In this example, they still engage a considerable amount of cooperative play – outcome state 4 in Figure 4C is self-cooperate, other-defect *o*_4_ = {*u*^*s*^ = 1, *u*^*o*^ = 2}. These outcomes reflect the preference of *self*, not a lack of confidence or expected precision. The evolution of expected precision is interesting. Precision reflects whether the available policies can fulfill the goals or utilitarian priors. Initially, there was prior belief that fully cooperative play might be achieved, given the *other* might be prosocial. When it looked as if this was the case (outcome state 2 in Figure 4C), precision jumped optimistically (4D). However, overall, there is a slower increase in expected precision, as the agent realizes the true nature of the opponent (i.e., that the *other* is antisocial). This illustrative example highlights the important interplay between prior beliefs about outcomes, inference on hidden states or characteristics of opponents and, crucially, confidence in the ensuing beliefs.

## Discussion

In this paper, we applied active inference to interpersonal decision making. Using a simple example, we captured key aspects of single and repeated exchanges. This example belongs to the large family of partially observable Markov decision problems (POMDP) but its solution is distinguished by explicit consideration of the agent's goals as *prior* distributions over outcomes. Because behavior depends upon beliefs, this necessarily entails beliefs that have precision. In other words, it is not sufficient simply to consider the goals of interpersonal exchange, one also has two consider the confidence that those goals can be attained. We have focused on optimizing this precision of beliefs about different policies—as opposed to sensitivity to different outcomes. In what follows, we consider the difference between sensitivity and precision. We then consider the nature of interpersonal inference and how it shapes decision-making. Finally, we discuss further developments along these lines.

### Sensitivity over outcomes vs. precision over policy choice

One of the key consequences of our formulation is the separation of choice behavior into two components. The first is inherent in the prior distribution itself, which reflects goals that are not directly represented in the exchange—as might be codified by various matching rules or exploratory drives. The second is optimized by the agent during the exchange itself in order to maximize utility or returns, in light of what is realistic. As described in Friston et al. (2013), this decomposition can be seen clearly by expressing the negative divergence—that constitutes prior beliefs—in terms of entropy (promoting exploration of allowable states) and expected utility:

(9) $$-D_{KL}[P(s_{T}|s_{t},\;\tilde{u})||P(s_{T}|m)]=H[P(s_{T}|s_{t},\;\tilde{u})]+E_{P(s_{T}|s_{t},\;\tilde{u})}[\ln P(s_{T}|m)]$$

Therefore minimizing the difference between attainable and desired outcomes can always be expressed in terms of maximizing expected utility, under the constraint that the entropy or dispersion of the final outcomes is as high as possible.

This separation of choice behavior—into (context-sensitive) beliefs about policies vs. (context invariant) beliefs about which outcomes are desirable—is reflected by an introduction of precision *γ* to complement the softmax sensitivity β. Both parameters play the role of precision or sensitivity (inverse temperature). β determines how sensitive prior beliefs are to rewards or the relative utility of different outcomes. However, this does not specify the confidence or precision that these outcomes can be attained. This is where the precision parameter *γ* comes in—it encodes the confidence that desired outcomes can be reached, based on current beliefs about the world and allowable policies. For example, one can be very uncertain about the contingencies that intervene between the current state and final outcome, even if one is confident that a particular outcome has much greater utility than another.

Crucially, the precision of the probability distribution over alternative policies can itself be inferred in a Bayes-optimal sense. This represents a departure from classical formulations. It arises because we are formulating policy selection in terms of inference. Choices are based upon beliefs (or inference) and beliefs in turn are held with greater or lesser confidence. The Bayes-optimal selection of precision over policies is a key thing that the current formulation brings to the table, above and beyond classical formulations.

### Interpersonal representations as motivating beliefs

Our modeling demonstrates that the formulation of interpersonal representations in terms of plausible and desirable outcomes accommodates a number of psychological findings and points to interesting theoretical and empirical questions.

First, our model replicates basic features of other successful models of interactive games. The ‘esteem’ traits in our model parallel the role of fairness-related coefficients in other models (Xiang et al., 2012). Second, our model infers the type of the partner (e.g., Figure 4A) and adjusts its policy so that it is not exploited (Figure 4B). Thirdly, posterior beliefs are based upon a generative model that entails beliefs about beliefs (utility functions) of others. This endows the generative model with an elemental theory of mind. Furthermore, Bayesian inference about esteem, and therefore intentions, constitute an elementary form of *mentalizing* (Allen et al., 2008).

In our case the fact that interpersonal representations contribute to the agent's beliefs about the desirability of outcomes *biases inference about states perceived* and actions selected. The perceptual update in Figure 1 contains a contribution from precision. This is a remarkable effect of approximate Bayesian inference. In our example (Figure 4B) the result is that the agent is biased toward co-operativity, despite believing that the *other* is as likely to be uncooperative as not (Figure 4A). This is an interpersonal analog of optimism bias, or ‘giving the benefit of the doubt’. There is experimental evidence in the Trust task that beliefs about prosocial traits in the *other* result in preference structures akin to the prosocial side of Figure 2A. When Investors are made to believe that the Trustee is of ‘moral character’ they entrust larger amounts (in our terms, cooperate in a sustained manner) even if the experimenter manipulates Trustee behavior so that the Investor does not make more money as a result (Delgado et al., 2005).

Our treatment suggests that interpersonal representations can help predict (and seek out) the outcomes of interactions. The idea that a self-esteem aspect of self-representation helps predict social outcomes is a central empirical finding of research by Leary and co-workers (Leary et al., 1995). Aspects of other-representation that help predict active social outcomes can be found in negative ideas about others, that healthy people harbor in certain contexts. As mentioned, exaggerated suspicion about others can serve to manage false-negative errors in the detection of social difficulties (Kramer, 1994). Computationally, more sophisticated agents can predict interactions better. Under certain constraints, however, interpersonal beliefs in the form of prosocial biases help achieve behavior that emulates such sophisticated thinking, a key theoretical finding of Yoshida and co-workers (Yoshida et al., 2008).

Interpersonal inference suggests that the use of self-representations to predict outcomes requires an *assessment of context*. In our Trust task, my partner and I can just consider one round in the future, provided we have inferred our types appropriately and, implicitly, the effective nature of the exchange (cooperative or competitive, etc.). Our simulation contains an interesting example of what happens if the wrong representations are assumed. The game is cooperative but, in our example, the other is antisocial (and unsophisticated). The *other*'s preference, stemming from their negative “niceness” (esteem), is to defect while the *self* cooperates, followed by mutual cooperation. Note that this preference structure is the only element in our naïve *other*'s cognitive machinery. When the *self* infers this preference structure they switch to a more uncooperative policy, thus undermining the *other*'s goals. Had the *other* been “nice” enough, or had they believed the *self* to be “nice” enough, the *self* would have inferred this and the *other*'s predictions, or goals, would be fulfilled.

We see that goals are not prescribed by immediate reward but by more generic beliefs. Clearly, there are an enormous number of forms for these beliefs that we could consider that help predict and realize different outcomes in different contexts. In the present context, one might consider the long-term payoffs that accrue from a collaborative policy for the agent or for everybody. Crucially, collaboration entails a consilience in terms of prosocial preferences or utility. The key thing about prosocial utility is that it can be symmetrical with respect to me and my opponent. For example, I may altruistically value the total reward accrued by myself and my opponent *if* I think they are prosocial, but only my own rewards if they are antisocial. In our simple illustration, and with the right choice of parameters, this would result in a very similar pattern of exchange to that seen in Figure 3. Alternatively, through some aversion to inequality, *self* might prefer equitable outcomes (irrespective of who gets most).

In our simulations the effect of esteem operates like a *social Pavlovian bias*, biasing beliefs irrespective of their consequences. A Pavlovian bias enhances certain actions in certain contexts. For example, it enhances passivity in a context of threat or vigorous approach in a context of opportunity, irrespective of instrumental outcomes. Our social Pavlovian bias promotes certain actions in the context of certain personal esteems irrespective of instrumental outcomes. Here, we chose a scheme of social Pavlovian biases that makes direct links between contemporary research into these fundamental biases (Guitart-Masip et al., 2012) and the large body of clinical- and social- psychological work on affectively charged representations of people. This work spans Aristotelian ethics, forensic psychotherapy (Gilligan, 2000) through to attribution theory (Thewissen et al., 2011).

We placed emphasis on prior beliefs as they may absorb various beliefs about long-term outcomes. These utilitarian beliefs entail the agents' cognitive-affective horizon, beyond which the agent has no knowledge and no control. This contrasts with the dynamics of the exchange, wherein the agent has both beliefs about states and beliefs about control. We envisage that the present approach will help disentangle these two components in the setting of interpersonal dynamics.

Although our ultimate aim is to study how self-representation is inferred under active inference, in this introductory study we have kept self-representation constant. Although we hope to examine this in future work here we note that a Bayesian framework naturally predicts that ordinary self-representation should be less responsive to evidence than the representation of others. Setting aside beliefs about changeability of the self, as well as the real possibility that aspects of self-representation may be learnt “once and for all” during childhood, inference about self-representations must take place on the basis of a much greater evidence base than inference about strangers. Therefore each new piece of evidence is expected to have less impact on self-representation than other-representation.

### Modeling choices, limitations and outlook

#### What does my partner think of me?

It may appear that we made a gross simplification in modeling the *self* using their own representation to estimate how the *other* sees the self. A more general formulation might be more conventional, where the beliefs of the *self* (self-representation and reputation with respect to others) are separate. Yet this is not a modeling choice made to make the model simpler. For example, clinical psychology indicates that beliefs about the *self* are highly correlated with beliefs about how others see the *self*. Moreover, patients with unwarranted beliefs about themselves and others that look “psychologically defensive” show no greater social desirability than healthy controls (Moutoussis et al., 2013). We suggest that the *self* uses beliefs about their esteem to model the beliefs of the *other*, a generalization of the “sociometer” theory with a view to testing the limits of this assumption's predictive power.

#### Depth-of-thought

Our model uses a very simple *other*, who makes no inferences about itself. Clearly, this is not a realistic simulation of *other*. Furthermore, our model *self* does did not explicitly calculate distant outcomes before applying the prior “horizon.” The latter is partly justified as most people look to the future to quite a limited extent. In the Trust Task, only about a quarter of Investors show up to two levels of recursive interpersonal thought (Xiang et al., 2012). Having said this, further work needs to consider agents that explicitly simulate outcomes for a small number of steps into the future and apply inference and preferences to patterns of such outcomes.

#### Normative self-representations

We envisage that self representations would enter into the assessment of proximal gains in the light of long-term outcomes; for example, “What sort of person am I, if I treat the other player like this?”; “If that's the sort of person I am, how am I likely to be treated in the future?” This extension of the simple model above will be crucial if the *other* makes inferences about the *self*. Our long-term aim, test the hypothesis that the normative role of self-representation is to predict the likely outcomes of social interactions, is likely to require such complex thinking. We envisage that beliefs about the opponent can, through conditional dependencies among Bayesian estimates about me and my opponent, affect beliefs about me. This may be crucial for understanding psychopathology in interpersonal exchange.

#### Model parameterization

We discussed above that interpersonal, affectively charged representations may be parameterized in a number of related ways. We chose a very simple parameterization for the purposes of demonstration. In the light of a wider literature, the validity of different models for interpersonal representation and the relationships between them remain to be clarified. One important contribution of formal models, of the sort we have introduced here, is that they can provide quantitative predictions of choice behavior. In principle, this means that one can use observed choices to estimate the parameters of a given model and—more importantly—use Bayesian model comparison to adjudicate between different forms or hypothetical schemes.

## Summary

In conclusion, we have sketched an elementary model of *self* and *other* representation during interpersonal exchange; within which these representations have important functional roles. We have seen that it is fairly straightforward to place optimal decision schemes in an active inference framework. This involves replacing optimal policies, defined by utility functions, with prior beliefs about outcomes. The advantage of doing this is that one can formulate action and perception as jointly minimizing the same objective function, which provides an upper bound on surprise or (negative log Bayesian) model evidence. This enables optimal control to be cast as a pure inference problem, with a clear distinction between action and inference about (partially) observed outcomes. Using a simple example, we have demonstrated how desirable goals can embody and express prosocial preferences as well as beliefs about the type of an opponent. Specifically, we have shown how these beliefs can be updated during iterated play and how they can guide interpersonal choices. Although rudimentary, these simulations illustrate a formal basis for interpersonal inference.

## Author contributions

Michael Moutoussis formulated the core hypotheses regarding interpersonal representation from both a clinical-psychological and a Bayesian perspective; formulated the test task and its parameterization; programmed the structure of the task; ran the simulations; and drafted much of the manuscript. Raymond J. Dolan provided supervision to Michael Moutoussis; provided management for the whole project; provided psychiatric expertise; and edited the manuscript. Nelson J. Trujillo-Barreto and Wael El-Deredy provided help with variational Bayesian modeling; and reviewed the manuscript. Karl J. Friston placed the hypotheses regarding interpersonal Bayesian inference within the framework of Active Inference; contributed heavily to the mathematical formulation; wrote and provided key code within the SPM environment for solving discrete partially observable Markov problems through the message-passing free-energy-minimization algorithm; edited the manuscript; and provided supervision to Michael Moutoussis.

### Conflict of interest statement

The authors declare that the research was conducted in the absence of any commercial or financial relationships that could be construed as a potential conflict of interest.

## Footnotes

^1^Here we present the model step-by-step; see also the Discussion section regarding the rationale behind specific modeling choices.
